# A study on the effects of the Qihuang Needle therapy on patients with Parkinson's disease

**DOI:** 10.3389/fneur.2022.1022057

**Published:** 2023-01-27

**Authors:** Xinyu Li, Jingpei Zhou, Renxiu He, Jiahui Lian, Jie Jia, Chialin Hsu, Shihua Yuan, Zhenhu Chen

**Affiliations:** ^1^Medical College of Acupuncture, Moxibustion, and Rehabilitation, Guangzhou University of Chinese Medicine, Guangzhou, Guangdong, China; ^2^The First Affiliated Hospital of Guangzhou University of Chinese Medicine, Guangzhou, Guangdong, China; ^3^Baiyun Branch of Nanfang Hospital, Southern Medical University, Guangzhou, Guangdong, China; ^4^The Affiliated Traditional Chinese Medicine Hospital, Guangzhou Medical University, Guangzhou, Guangdong, China; ^5^Department of Ultrasound, The First Affiliated Hospital, Guangzhou University of Chinese Medicine, Guangzhou, Guangdong, China; ^6^International College, Guangzhou University of Chinese Medicine, Guangzhou, Guangdong, China

**Keywords:** acupuncture, Qihuang Needle therapy, Parkinsion's disease, randomized clinical trial, sham acupuncture

## Abstract

**Objective:**

This study aimed to evaluate the effectiveness of the Qihuang Needle (QHN) in treating Parkinson's disease (PD).

**Design, setting, and participants:**

The trial was an 8-week randomized clinical trial (4 weeks of treatment followed by 4 weeks of follow-up) conducted from January 2021 to July 2022 in outpatient settings at three clinical sites in Guangzhou, China. Thirty-four participants with PD were diagnosed based on the diagnostic criteria formulated by the brain bank of the British Parkinson's Disease Society in 1992.

**Interventions:**

Patients in the treatment and control groups received six sessions within 4 weeks of the QHN therapy or the sham acupuncture therapy (two times per week for the first two consecutive weeks and one time per week for the following two consecutive weeks).

**Main outcomes and measures:**

The primary outcome measure was the change in the Parkinson's Disease Rating Scale-Part III Motor Examination (UPDRS III) between baseline and 8 weeks after treatments. Secondary outcome measures were the Non-Motor Symptoms Scale for Parkinson's Disease (NMSS) and Parkinson's Disease Daily Quality of Life-39 (PDQ-39). Real-time shear wave elastography (SWE) was assessed for each patient at baseline and during the 4-week period as the third outcome measure.

**Results:**

A more significant reduction of UPDRS III score, PDQ-39, NMSS, and SWE was observed in the QHN group than in the sham acupuncture group.

**Conclusions:**

The QHN therapy consistently demonstrated superiority and produced clinically meaningful benefits in reducing motor and non-motor symptoms, as well as significantly improving muscle stiffness, in patients with PD.

## Introduction

Parkinson's disease (PD) is a progressive neurodegenerative disease characterized by tremors and bradykinesia and is a common neurologic ailment. The appearance of motor symptoms accompanies the diagnosis of PD. Specific non-motor symptoms, such as olfactory dysfunction, cognitive impairment, mental symptoms, sleep disorders, autonomic dysfunction, pain, and fatigue, characterize the prodromal stage of PD. Axial movement symptoms, such as frequent falls and postural instability with a frozen gait, often occur in the advanced stages of the disease. As the second most common neurodegenerative disease, the incidence of PD before the age of 50 is low. However, it increases rapidly with age, peaking in most studies around the age of 80 (1). More than 6 million individuals across the world have PD (2). Drugs that increase the concentration of dopamine in the brain or stimulate dopamine receptors are still the main drugs for treating the motor symptoms of PD. These drugs include levodopa, dopamine agonists, type B monoamine oxidase inhibitors, and amantadine. Unfortunately, these drug treatments for PD usually increase the risk of adverse events (3, 4), including nausea, daytime sleepiness and edema, and impulse control disorders. Due to the limitations of these conventional treatments, efforts have been made to identify practical, low-risk interventions.

In China and Western countries, acupuncture has been widely used to treat PD. Acupuncture and moxibustion can reduce the adverse reactions caused by anti-PD drugs and improve the quality of life of patients (5, 6). Acupuncture has been shown in previous studies to be an effective adjunctive therapy for the treatment of PD, particularly in improving motor and non-motor symptoms. Some studies even suggest that acupuncture may be more effective than western medicine alone in treating PD (7–9).

Qihuang Needle (QHN) therapy is a treatment method developed by Professor Zhenhu Chen from the First Affiliated Hospital of Guangzhou University of Chinese Medicine that combines manipulation with acupuncture. It is based on the principles of tendon differentiation and uses modern anatomical theory to simplify the selection of points, resulting in a treatment that is simple, safe, and effective with fewer points required.

The primary purpose of this randomized, controlled, evaluator-blind trial study was to evaluate the effectiveness of QHN therapy as an adjunctive treatment for PD.

## Methods

### Study population and protocol

Participants in this study were recruited through advertisements on bulletin boards in the acupuncture and neurology departments at three hospitals: the First Affiliated Hospital of Guangzhou University of Chinese Medicine, the Guangdong 999 Brain Hospital, and the Third Affiliated Hospital of Sun Yat-sen University. An independent assessor working in these departments screened and registered participants who met the inclusion criteria. PD was diagnosed using the diagnostic criteria established by the brain bank of the British Parkinson's Disease Society in 1992 (10). The participants were recruited from January 2021 to July 2022. The Clinical Trial Center study protocol at the First Affiliated Hospital of the Guangzhou University of Chinese Medicine approved the protocol (11). The published protocol is available at the following link: https://www.frontiersin.org/articles/10.3389/fneur.2022.902170/full.

The inclusion criteria were as follows: men or women aged 40–80 years with a diagnosis of PD; patients with experience with PD for at least 1 year; patients whose Hoehn-Yahr (HY) grades range from 1 to 4; patients who were on anti-PD medication and had been on a stable dose for more than 2 months or who had not been on medication for more than 2 months; stable vital signs and clear consciousness; and the provision of written, informed consent by the patients.

Patients with any of the following conditions were excluded: the presence of severe hepatorenal disease, tumors, bleeding disorders, endocrine disease, or infection; schizophrenia or other mental disorders that affected compliance; being deaf or having communication difficulties caused by dementia; having a history of alcohol or drug abuse; or involvement in other clinical trials.

### Randomization and blinding

A total of 34 eligible patients were recruited and randomly assigned at a ratio of 1:1 to receive QHN or sham acupuncture (SA) treatment. The Clinical Medical College of Acupuncture, Moxibustion and Rehabilitation, Guangzhou University of Chinese Medicine, Guangdong Province, China performed the central randomization. The random assignment operation was programmed and executed using the SAS9.2 software. An independent researcher received the random numbers and group assignment after inputting the patients' information through the application. Participants in the treatment group (QHN group) and the control group (SA group) were blinded. Acupuncturists could not be blinded to the treatment assignments, given the nature of the interventions. Outcome assessors, data collectors, and statisticians were blinded to the treatment allocation.

### Intervention

Patients in the treatment and control groups received six sessions within 4 weeks of QHN or SA therapy (two times per week for the first two consecutive weeks and one time per week for the following 2 weeks) for 4 weeks. Five to six acupoints were used per treatment, located in the four limbs, the neck, and the back. We chose the prescriptions as a result of our experience from our previous study (11). All acupuncturists were trained and licensed with at least 5 years of clinical experience.

Patients in the treatment group were treated with QHN therapy. A tailored, sterile, stainless-steel needle (length: 50 mm; diameter: 0.5 mm; QH; Chongqing) was inserted into the described acupoints at a depth of 25–40 mm. After the patients felt the Deqi sensation, the needles were removed and reapplied at a 30° angle. All needles were withdrawn with clean cotton balls pressed to the skin to prevent bleeding. In addition, more details of the procedure have been published (11).

The number of needles and duration of treatment in the control group were identical to those in the treatment group except that an attempt was not made to induce the Deqi sensation. The control group had undergone insertion of QHN at sham acupoints that did not correspond to acupuncture points. Sham acupoints were defined as 20 mm lateral to the real acupoints. Therefore, a needle was inserted into the sham acupoints at a depth of 3 mm without any subsequent manipulation. At these points, the patient did not feel any Deqi sensation.

To ensure the safety and compliance of participants and meet ethical requirements, we followed the recommendations for managing patients with PD as outlined in the Chinese guidelines (10).

During the treatment period, two groups of patients were orally administered anti-PD drugs. The subjects taking the drugs were responsible for preparing and maintaining their original treatment regimens. In instances where a change in medication was necessary, we carefully recorded details such as the name of the medication, time of administration, and dose.

In addition, frozen gait (FOG) is one of the most disabling gait disorders, affecting 80% of patients with PD. Clinical guidelines recommend that these patients have gait rehabilitation as soon as possible (12). Some studies (13–15) also demonstrated that gait rehabilitation has immediate real-life benefits on FOG symptoms among patients with mild-to-moderate PD. Therefore, subjects were instructed to perform home-based rehabilitation training according to Parkinson's easy quintile, one time in the morning and one time at night. The five pieces of the brief parkinsonian movement were designed and guided by Professor Zhenhu Chen and included *in situ* steppings, left-right translation, finger percussion, head massage, and abdominal massage.

### Measurements

#### Data collection

All outcome measurements were taken at baseline (before treatment), 4 weeks after, and 8 weeks after treatment. At each follow-up, two blinded evaluators at each clinical center reminded patients by phone or text message to return the headache diary to the trial offices *via* email or to outpatient offices at follow-up visits.

#### Outcome measures

The primary outcome was the change in the PD Rating Scale-Part III Motor Examination (UPDRS III) between baseline and 8 weeks after randomization. Secondary outcome measures included the Non-Motor Symptoms Scale for PD (NMSS) and the PD Daily Quality of Life-39 (PDQ-39). In addition, real-time shear wave elastography (SWE) was assessed at baseline and every 4 weeks.

#### Sample size calculation

This trial used a clinical-superiority design to verify that QHN therapy's effect was superior to SA. The primary outcome measure was the UPDRS III score difference before and after the treatment. According to previous research (16), we assumed the standard deviation (SD) to be 8.0 and the mean of the treatment effect of the two groups to be 4.36 and 0.25, respectively, during which the statistical power was 80%, and the significance level was 0.05. Using PASS software meant each group had to contain more than 17 patients.

### Statistical analysis

We used mean and SD for normally distributed variables or median (interquartile range) for the variables not normally distributed to summarize the participants' demographics, health conditions, and clinical outcomes at two different time points. Two statisticians, blinded to the group setting, analyzed the data independently *via* SPSS software (version 23) and R (version 4.1.0). Furthermore, the data were analyzed using the intent-to-treat principle. The normality of the variables was assessed using the standard probability plot. The continuously distributed variables were customarily assessed by the student's *t*-test. Otherwise, the Mann–Whitney or Wilcoxon test was applied. Fisher's exact test was adopted for categorical data, and statistical significance was set at a *p*-value of < 0.05.

## Results

### Demographic and clinical characteristics

**Figure 1:**
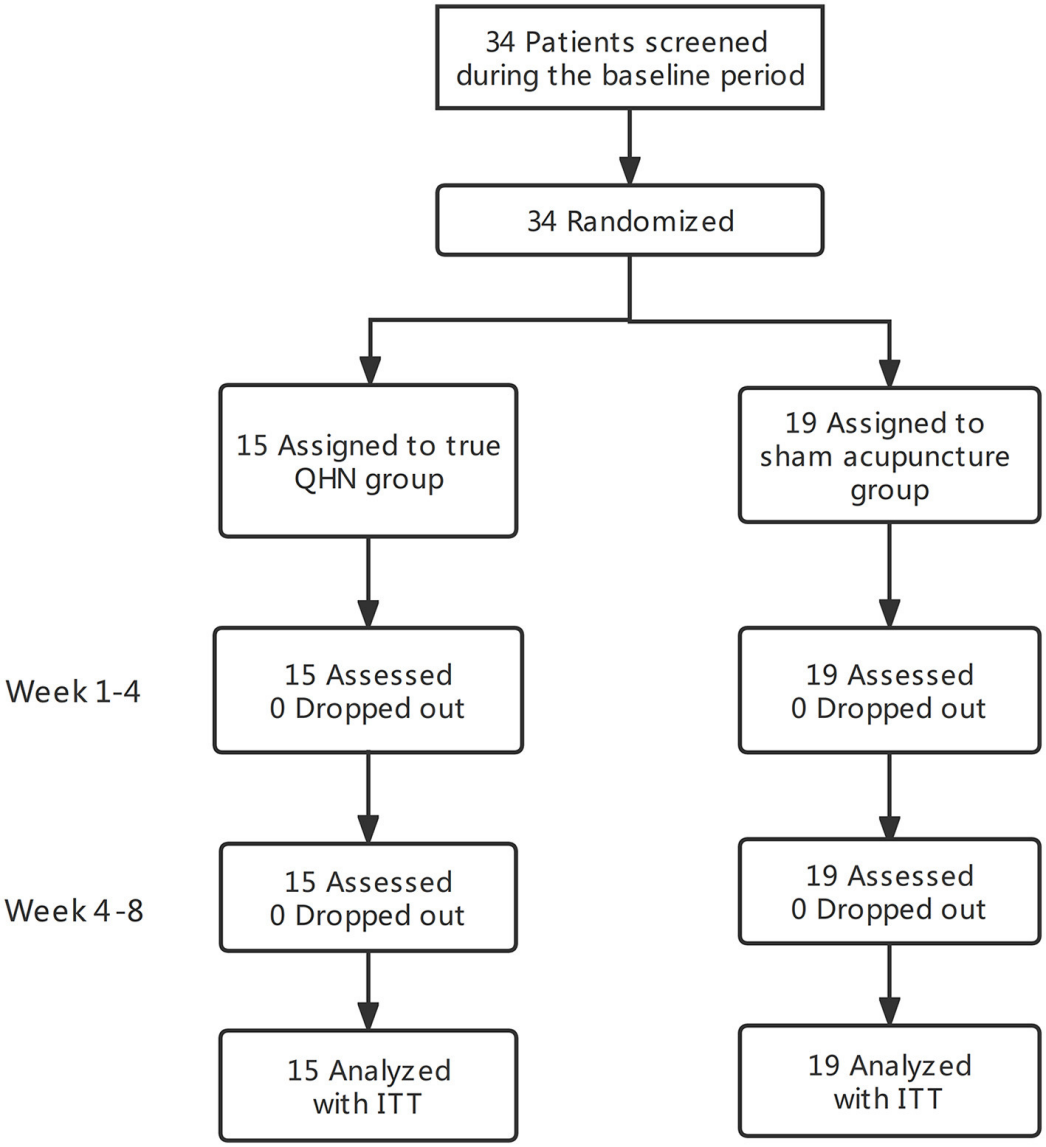
A flowchart of the screening, enrollment, randomization, and follow-up of this study.

Thirty-four participants, 40–80 years old, were randomized. A total of 55.8% were women. Fifteen patients in the QNH group and 19 in the SA group were separated from 34 patients with PD. Intent-to-treat (ITT) analysis was conducted on the two groups. The baseline characteristics did not differ significantly between the groups. All participants completed the assessments without the occurrence of significant adverse events. The flowchart of this study is shown in Figure 1.

### Primary outcome

**Figure 2:**
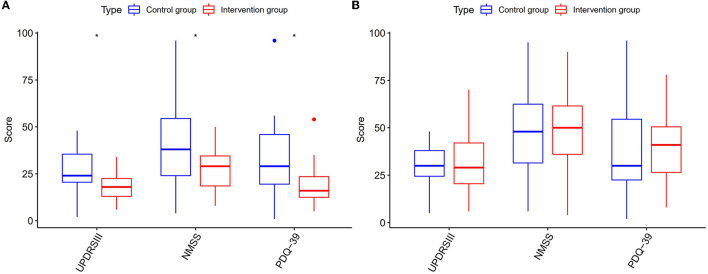
Multi-evaluation score (UPDRS III, NMSS, and PDQ-39) of PD status. Intervention group: QHN group. Control group: SA group. **(A)** The evaluation scores before treatment between two groups. **(B)** The evaluation scores after treatment between two groups.

Before treatment, the UPDRS III myotonia scores for both groups did not follow a normal distribution. We used the Mann–Whitney *U* test, a non-parametric test for independent samples, to compare the scores between the two groups and found that there was no statistically significant difference, with a *p*-value of 0.477 > 0.05. This indicates that the baseline scores for the treatment group and the control group were similar, allowing for meaningful comparison. The Wilcoxon signed rank sum test, a non-parametric test for paired samples, was used to compare the scores before and after treatment within each group. The treatment group showed a statistically significant difference, with a *p*-value of 0.00 < 0.05, as did the control group, with a p-value of 0.00–0.05. These results suggest that both groups had significant changes in UPDRS III myotonia scores following treatment. After treatment, the Mann–Whitney *U* test was used to compare groups. From Figure 2B, the *P*-value was determined to be < 0.01. The difference in the UPDRS III myotonia score between the two groups after treatment was statistically significant. The UPDRS III decreased in the QHN group by 15. A more significant reduction was observed in the QHN group than in the SA group.

### Secondary outcomes

Furthermore, the effects of acupuncture on the secondary outcomes seemed to be consistent during the follow-up (Figures 2A, B). In patients with PD of the QHN group, the score of the NMSS decreased by 11, and the score of the PDQ-39 decreased by 19 than before treatment. At the same time, we observed that NMSS and PDQ-39 were significantly reduced in the QHN group compared with the SA group, and the two groups had significant differences.

**Figure 3:**
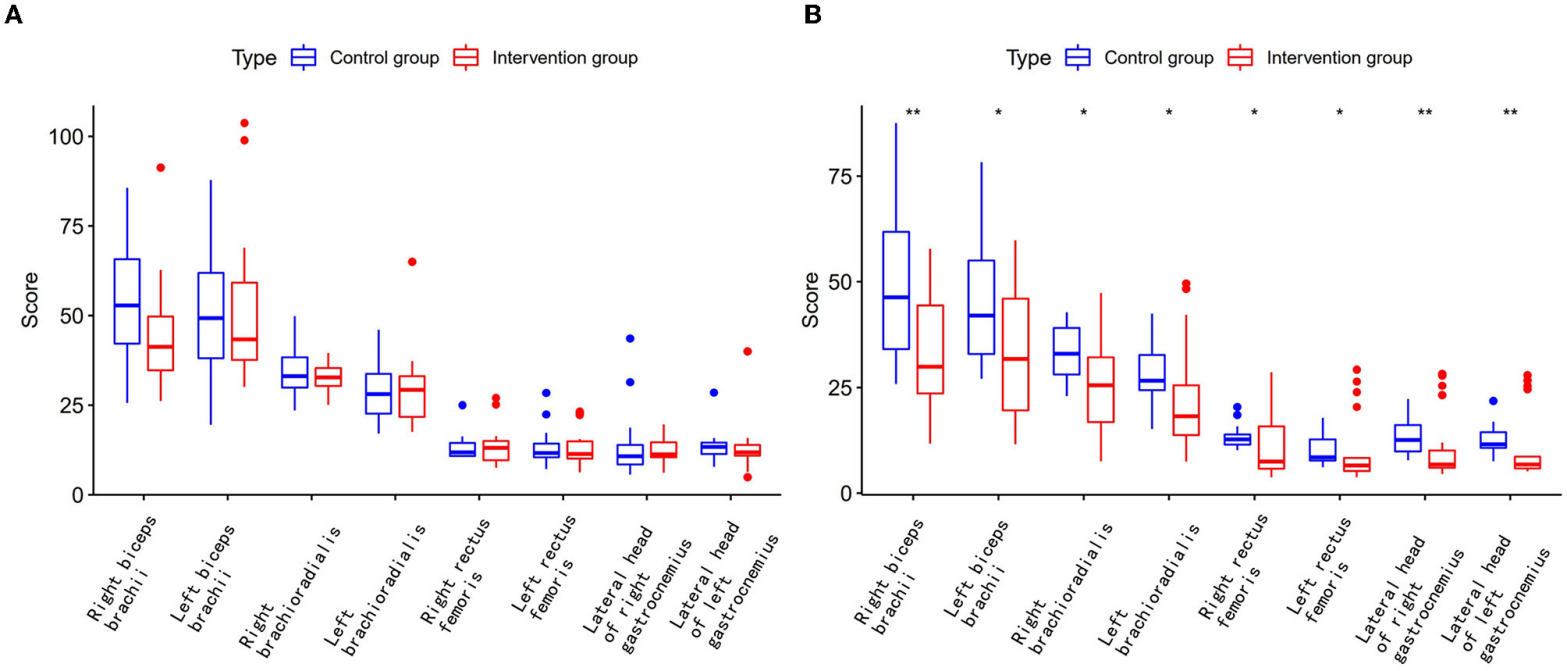
Real-time shear wave elastography score of PD patients. Intervention group: QHN group. Control group: SA group. **(A)** The SWE score before treatment between two groups. **(B)** The SWE score after treatment between two groups.

Moreover, the total score of SWE was significantly lower in the QHN group than in the SA group in each interview during weeks 3–6 (Figures 3A, B). We found that the SWE score for the right biceps brachii, the left biceps brachii, the right brachioradialis, the left brachioradialis, the right rectus femoris, the left rectus femoris, the lateral head of the right gastrocnemius, and the lateral head of the left gastrocnemius were significantly decreased before QHN treatment. It was comparable with the SA group, which illustrated that QHN had a superior efficacy on the tension situation of patients' body muscles and joints to achieve greater freedom of movement. We observed significant differences in the SWE score between the two groups at weeks 2, 3, and 4 of the follow-up period. The QHN group showed a reduction in patient discomfort during treatment compared to the SA control group.

## Discussion

In our study, we investigated whether a 4-week QHN treatment improved activities of daily living and motor symptoms in patients with PD. We obtained vital evidence to help interpret previous clinical trials that studied the effects of QHN treatment on patients with PD. In psychophysical responses, the UPDRS III, NMSS, PDQ-39, and SWE were statistically lower after acupuncture treatment for 4 weeks, and the UPDRS III, NMSS, and PDQ-39 remained stable for at least the next 8 weeks (8-week follow-up). Decreases in these scores mean improvements in the symptoms related to these scores in patients with PD. In addition, after 4 weeks of treatment, the control group also showed significant changes compared to the baseline, and there were significant differences between the control and treatment groups. There were no serious adverse events due to the treatment. These results indicate the safety and usefulness of the combined treatment of QHN for patients with PD as an adjunctive treatment.

There have been observations and research reports on the population of patients with PD (8, 17, 18), which confirmed the effectiveness of acupuncture and moxibustion in treating PD. These studies reported improvements in PD symptoms, especially in those with milder disease. Hong et al. (19) compared two groups, one treated with VR rehabilitation training and one treated with Jiao's scalp acupuncture. For 8 weeks, selected participants received acupuncture one time a day, five times a week. Outcomes reveal that acupuncture could improve gait parameters, walking ability, and motor function in patients with PD.

Second, SWE is a new ultrasound technique capable of measuring the shear wave velocity of tissues and calculating Young's modulus values reflecting this index to evaluate tissue elasticity. Real-time shear wave elastography uses the shear wave of an ultrasound probe to create pressure on tissues and avoid the influence of artifacts, in addition to the advantages of non-invasive, convenient, and reproducible detection (20). It has recently been shown that the upper limbs of patients with PD are mainly the flexor and adductor muscles with increased muscle tone, such as the biceps brachii. Yin et al. (21) found that the values of Young's modulus of the gastrocnemius medialis (GM) in the lower leg of patients with PD on the symptomatic side were higher than those of healthy individuals (*P* < 0.05) and also higher than those on the less symptomatic side (*P* < 0.05). Clinically, PD muscle tone rises gradually, so generalized muscle stiffness often appears in the end stage of the disease. There are also findings suggesting that shear wave elastography in patients with PD also presents this feature, as Ding et al. (22) found that the shear wave velocity of the biceps brachii on the ankylosed side, the non-ankylosed side, and the brachioradialis on the tonic side in patients with PD was correlated with disease duration (*P* < 0.05).

The QHN body is hollow, and the needle's hardness is high, which strengthens the stimulation of acupuncture and thus improves clinical efficacy. Based on the theory of meridians and tendons, the acupoints for treating PD by QNH therapy mainly select the tendons near the joints. Relevant studies (23) found that acupuncture of tendon nodes can quickly relieve the spasticity of local soft tissues, accelerate blood circulation, increase local tissue nutrition, and finally improve the related symptoms of patients with high muscle tension rapidly, such as muscle stiffness and pain. In selecting needling methods, QNH therapy inherits the five needling methods of classical needling. It is recorded in *Su Wen Ji Zhu that the Qi of the five viscera is external to the skin, veins, muscles, and bones, and the five viscera are in the middle*, so the external combination should be applied to the five viscera. It can be seen that the five-needling method has a targeted therapeutic effect. The location of PD involves the liver, the spleen, the kidney, the muscles, the tendons, and the bones. Therefore, the clinical operation is mainly based on Guan-needling, Hegu-needling, and Shu-needling.

Currently, many clinical studies and animal experiments show that acupuncture has an excellent effect on PD without apparent toxic or side effects. Relevant literature (24) pointed out that the mechanism of acupuncture treatment for PD includes promoting the expression of neurotrophic factors in the brain, reducing abnormal metabolites in the brain, reducing the aggregation of a-synuclein, inhibiting neuronal apoptosis, inhibiting oxidative stress, inhibiting endoplasmic reticulum stress, and regulating intestinal flora. The pathogenesis of PD is complex, but the current acupuncture research only starts from a specific mechanism and fails to comprehensively explain the relationship between various mechanisms and what mechanism plays a leading role (25). QHN therapy is a new acupuncture method. There are few studies on the mechanism of QHN therapy in treating PD, and its therapeutic mechanism is unclear. However, the clinical effect of this therapy is accurate, and the prospects are good. It is worth further exploring its mechanism.

### Limitations

Our study also has three limitations: a small sample size of only 34 subjects due to the effects of COVID-19, the study's duration, and human constraints. The subjects were mainly from Guangzhou City, which may limit the generalizability of the results and impact the study findings as well as the short follow-up period of this study, preventing us from observing the long-term effectiveness of QHN therapy for treating PD.

## Conclusion

For at least 8 weeks, QHN therapy demonstrated persistent superiority and clinically relevant benefits in Parkinson's disease, including a reduction in motor and non-motor symptoms and a significant improvement in muscle stiffness.

## Data availability statement

The raw data supporting the conclusions of this article will be made available by the authors, without undue reservation.

## Ethics statement

The studies involving human participants were reviewed and approved by the Clinical Trial Center study protocol at The First Affiliated Hospital of Guangzhou University of Chinese Medicine. The patients/participants provided their written informed consent to participate in this study.

## Author contributions

XL designed and analyzed the research study. XL and JZ wrote and revised the article. RH, JL, CH, SY, and JJ collected and analyzed the data. All authors have read and approved the article.
